# Small molecules promote CRISPR-Cpf1-mediated genome editing in human pluripotent stem cells

**DOI:** 10.1038/s41467-018-03760-5

**Published:** 2018-04-03

**Authors:** Xiaojie Ma, Xi Chen, Yan Jin, Wenyan Ge, Weiyun Wang, Linghao Kong, Junfang Ji, Xing Guo, Jun Huang, Xin-Hua Feng, Junfen Fu, Saiyong Zhu

**Affiliations:** 1 Life Sciences Institute, Zhejiang University, 310058 Hangzhou, China; 2 The Children’s Hospital, Zhejiang University School of Medicine, 310003 Hangzhou, China; 3 Stem Cell Institute, Zhejiang University, 310058 Hangzhou, China

## Abstract

Human pluripotent stem cells (hPSCs) have potential applications in biological studies and regenerative medicine. However, precise genome editing in hPSCs remains time-consuming and labor-intensive. Here we demonstrate that the recently identified CRISPR-Cpf1 can be used to efficiently generate knockout and knockin hPSC lines. The unique properties of CRISPR-Cpf1, including shorter crRNA length and low off-target activity, are very attractive for many applications. In particular, we develop an unbiased drug-selection-based platform feasible for high-throughput screening in hPSCs and this screening system enables us to identify small molecules VE-822 and AZD-7762 that can promote CRISPR-Cpf1-mediated precise genome editing. Significantly, the combination of CRISPR-Cpf1 and small molecules provides a simple and efficient strategy for precise genome engineering.

## Abstract (web summary)

Precise genome editing in human pluripotent stem cells requires the development of methods for rapid and efficient genetic manipulation. Here, the authors screen for small molecules that enhance CRISPR-Cpf1-mediated genome engineering.

## Introduction

Human pluripotent stem cells (hPSCs), including human embryonic stem cells (hESCs) and human induced pluripotent stem cells (hiPSCs), offer a promising solution to study human early development and investigate human diseases. It is of paramount importance to develop methods for rapid, efficient, and controllable genetic manipulation of hPSCs^1,2^. Site-specific nucleases (SSNs) can induce double-strand breaks (DSBs) at desired genomic loci and trigger the endogenous DNA repair machinery. Processing of DSBs by non homologous end joining (NHEJ) pathway leads to small insertions and deletions (Indels) useful for generating knockout mutants, whereas homology-directed repair enables the generation of knockin mutants or reporter cell lines^3,4^. Even assisted with these SSNs, the precise genome editing in hPSCs remains very challenging.

Recently, CRISPR-Cpf1 has been identified^5^. CRISPR-Cpf1 recognizes thymidine (T)-rich protospacer adjacent motif (PAM) sequences (TTTN), expanding the range of RNA-guided genome editing; Cpf1 creates 5-nt staggered ends, which potentially initiate distinct DNA repair processes; the Cpf1 crRNA length is much shorter than that of Cas9, making it easier for in vitro synthesis and more suitable for multiplexed genome editing; the off-target activity of Cpf1 is low, which is desirable for precise genome editing^6–8^. CRISPR-Cpf1 not only provides an alternative method for targeted mutagenesis, but also greatly enhances the scope and precision of genome editing. However, whether CRISPR-Cpf1 can be used to do precise genome editing in hPSCs is largely unknown.

Chemical strategies have great applications in stem cell biology and regenerative medicine^9^. Several small molecules have been identified to modulate CRISPR-Cas9-induced genome editing^10^. Yu et al. identified L755507 and Brefeldin A that could enhance CRISPR-Cas9-mediated genome editing^11^. Chu et al. and Maruyama et al. found that the ligase IV inhibitor SCR7 could improve the efficiency of CRISPR-Cas9-mediated genome editing^12,13^. Because of the low efficiency of knockin in hPSCs, it is challenging to carry out a high-throughput chemical screening to identify small molecules that can promote CRISPR-Cpf1-mediated genome editing in hPSCs. Since the distinct properties of CRISPR-Cpf1 from CRISPR-Cas9, we are interested in establishing a feasible chemical screening system and identifying effective small molecules for precise genome editing in hPSCs.

Here, we demonstrate that CRISPR-Cpf1 can be used to efficiently generate knockout and knockin hPSC lines. Through chemical screening, we have identified two interesting small molecules VE-822 and AZD-7762 that enhance CRISPR-Cpf1-mediated precise genome engineering. The combination of CRISPR and small molecules holds great potentials in many applications.

## Results

### Generation of knockout hPSC lines using CRISPR-Cpf1

**Fig. 1:**
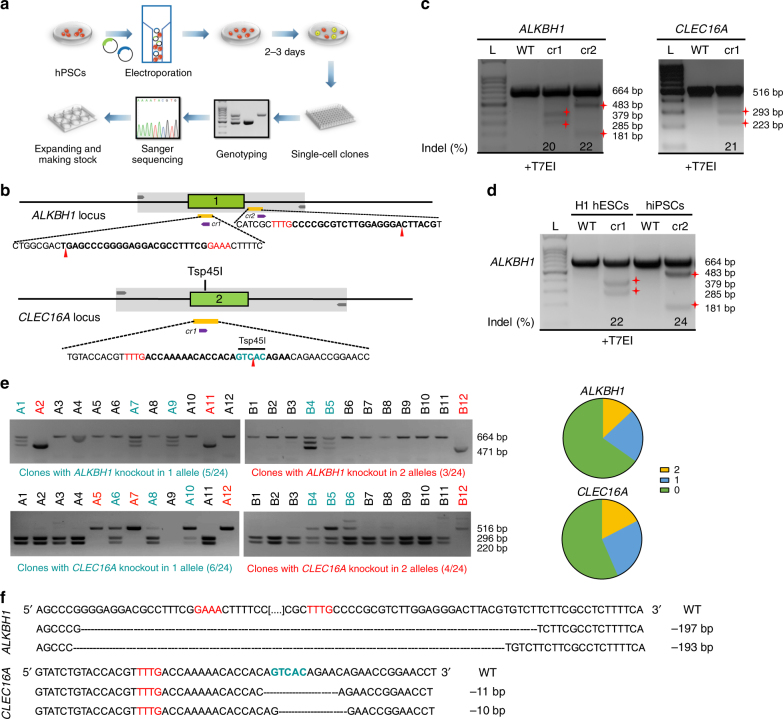
Efficient generation of knockout hPSC lines using CRISPR-Cpf1. **a** A scheme of the experimental procedure for generating knockout hPSC lines. **b** Schematic of Cpf1 crRNA targeting sites at *ALKBH1* and *CLEC16A* loci showing exon structures (green boxes), PCR amplicons (light gray boxes), and restriction sites used for PCR analysis. crRNA targeting sequences are in bold; PAM sequences are in red. **c** T7EI assay for crRNAs of *ALKBH1* and *CLEC16A* in MEL1 hESCs. The Indel frequency was calculated using the expected fragments. **d** T7EI assay for crRNAs of *ALKBH1* in H1 hESCs and hiPSCs. The Indel frequency was calculated using the expected fragments. **e** PCR analysis upon crRNA transfection. For *ALKBH1*, two crRNAs were transfected together. Clones with gene knockout in one allele are in blue, and clones with gene knockout in two alleles are in red. More detailed description and explanation of the band pattern can be found in Supplementary Fig. 2. **f** Sequencing results of the targeted allele in *ALKBH1* and *CLEC16A* knockout hPSC lines. PAM sequences are in red. Restrictive enzyme site is in blue

To develop the CRISPR-Cpf1-mediated genome editing system in hPSCs, we constructed a plasmid with a U6 promoter-driven crRNA expression cassette (Supplementary Fig. 1a, b). We picked several genes, including *ALKBH1* and *CLEC16A*, which we are interested in. ALKBH1 has recently been identified as a tRNA demethylase^14^. CLEC16A plays important roles in the development of diabetes^15^. To construct gene-specific crRNA plasmids, we used an online software (http://chopchop.cbu.uib.no) to design a panel of crRNAs specifically targeting these genes (Fig. 1b and Supplementary Table 1). We tested the genome editing capacity of these crRNAs in 293T cells and observed 20–30% Indel rates based on the T7 endonuclease I (T7EI) assays (Supplementary Fig. 1c). We then investigated the capacity of CRISPR-Cpf1-mediated genome editing in hPSCs (Fig. 1a). Using T7EI assays, we observed 20–30% Indel rates for *ALKBH1* and *CLEC16A* in hESCs and hiPSCs, indicating the efficient hPSC genome editing capacity of CRISPR-Cpf1 (Fig. 1c).

To further establish knockout hPSC lines, we passaged the transfected hPSCs at low cell density, picked colonies, and analyzed hPSC lines by PCR genotyping for *ALKBH1* or RFLP assay for *CLEC16A* (Fig. 1a). For *ALKBH1*, we co-electroporated the following plasmid mixture: pcDNA3.1-hLbCpf1, pCpfcr-ALKBH1-crRNA1, and pCpfcr-ALKBH1-crRNA2 into hPSCs. If these two crRNAs worked successfully, there was about 190-bp deletion on *ALKBH1* locus (Fig. 1b and Supplementary Fig. 2a, b). Consistently, we observed a 471-bp band for homozygous *ALKBH1* knockout clones, two bands (471 and 664 bp) for heterozygous *ALKBH1* knockout clones, and a 664-bp band for wild-type clones (Fig. 1e). Interestingly, an additional band occurred between 471 and 664 bp in heterozygous clones (Fig. 1e). It was a hybridized band revealed by Sanger sequencing and T7EI cleavage assay (Supplementary Fig. 2c, d, e). For *CLEC16A*, we co-electroporated the following plasmid mixture: pcDNA3.1-hLbCpf1 and pCpfcr-CLEC16A-crRNA1 into hPSCs. The CLEC16A-crRNA1-targeting site contains the Tsp45I restrictive enzyme site (Fig. 1b). For wild-type clones, the Tsp45I restrictive enzyme site was intact, and there were two bands (220 and 296 bp). For homozygous *CLEC16A* knockout clones, the Tsp45I site was edited with Indels. Therefore, we could only obtain a larger band with 516 bp. For heterozygous *CLEC16A* knockout clones, three bands (220, 296, and 516 bp) could be obtained (Fig. 1e and Supplementary Fig. 2f, g). We further calculated the efficiency of heterozygous and homozygous knockout. For *ALKBH1*, 20.8% colonies were deleted at one allele, and 12.5% colonies were deleted at both alleles (Fig. 1e and Supplementary Fig. 2a, b, c, d). For *CLEC16A*, 25% colonies were deleted at one allele, and 16.7% colonies were deleted at both alleles (Fig. 1e and Supplementary Fig. 2e, f). Sanger sequencing results confirmed diallelic deletion at the target sites of *ALKBH1* and *CLEC16A* (Fig. 1f). Potential off-target sites were amplified by PCR and sequenced by Sanger sequencing. We did not detect off-target effect from four *ALKBH1* knockout lines (Supplementary Fig. 3a, b) and two *CLEC16A* knockout lines (Supplementary Fig. 4a, b). It should be pointed out that the current assay has limitations, and it will be interesting to apply whole-genome sequencing to fully address this issue in the future. Taken together, these findings clearly demonstrate that CRISPR-Cpf1 can be used for efficient generation of hPSC knockout lines.

### Chemical screening for CRISPR-Cpf1-mediated knockin in hPSCs

**Fig. 2:**
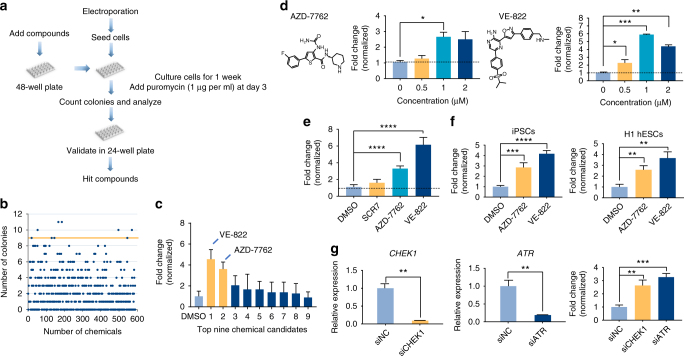
Identification of small molecules that can significantly promote CRISPR-Cpf1-mediated knockin in hPSCs. **a** A scheme of chemical screening. **b** The result of chemical screening. About 600 small molecules were screened, and the puromycin-resistant colony number was counted. Small molecules above the orange line were top chemical candidates for further confirmation. The average colony number of DMSO was 2. **c** The confirmation result of top small molecule candidates. VE-822 and AZD-7762 were the top two small molecules that increased the number of puromycin-resistant colonies. **d** The concentration tests of VE-822 and AZD-7762. *n* = 3 experiments. Statistical significance calculated using two-tailed Student’s *t*-test, compared to DMSO controls. *P < 0.05, **P < 0.01, ***P < 0.001. **e** The effects of VE-822 (1 μM), AZD-7762 (1 μM), and SCR7 (1 μM) in context of CRISPR-Cpf1-mediated knockin in hPSCs. *n* = 5 experiments. Statistical significance calculated using two-tailed Student’s *t*-test, compared to DMSO controls. ****P < 0.0001. **f** The effects of VE-822 and AZD-7762 were replicated in H1 hESCs and hiPSC lines. *n* = 4 experiments. Statistical significance calculated using two-tailed Student’s *t*-test, compared to DMSO controls. **P < 0.01, ***P < 0.001, ****P < 0.0001. **g** Knockdown of *ATR* and *CHEK1* by siRNAs and their effects on CRISPR-Cpf1-mediated knockin in hPSCs. *n* = 3 experiments. Statistical significance calculated using two-tailed Student’s *t*-test, compared to siNC controls. **P < 0.01, ***P < 0.001

To test the capacity of CRISPR-Cpf1 for generating knockin hPSC lines, hPSCs were co-electroporated with three plasmids: one expressing Cpf1, one containing a specific crRNA targeting *OCT4* which should be well designed^4^, and the knockin template containing an eGFP reporter and a puromycin-resistance cassette (Supplementary Fig. 5a)^16^. The transfected hPSCs were cultured for 2 days before subsequent puromycin treatment. After 4–5 days of 1 μg mL^−1^ puromycin selection, we observed several puromycin-resistant colonies. The efficiency of knockin is relatively low and needs to be further improved. However, we found that SCR7, which was reported to promote CRISPR-Cas9-mediated knockin, did not show any significant effect on CRISPR-Cpf1-mediated knockin (Fig. 2e). Thus, it is necessary and significant to identify chemical compounds for CRISPR-Cpf1-mediated knockin.

To improve the efficiency of CRISPR-Cpf1-mediated genome editing in hPSCs, we carried out a large-scale chemical screening using the OCT4-eGFP knockin with puromycin selection system (Fig. 2a). Briefly, small molecules were added into hPSC medium in 48-well plates before electroporation. Then hPSCs were co-electroporated with three plasmids: one expressing Cpf1, one containing the specific crRNA targeting *OCT4*, and the knockin template containing an eGFP reporter and a puromycin-resistance cassette. After electroporation, hPSCs were seeded into 48-well plates already added with small molecules. hPSCs were incubated with small molecules for 2 days and then administered with 1 μg mL^−1^ puromycin for another 4–5 days. The puromycin-resistant colonies were counted to quantify the functions of small molecules. From a collection of around 600 small molecules, we identified interesting small molecules that could increase the number of puromycin-resistant hPSC colonies (Fig. 2b). In the following confirmation testing, VE-822 and AZD-7762 were the two most effective compounds (Fig. 2c).

Then we focused on VE-822 and AZD-7762 for more detailed studies. We found that VE-822 and AZD-7762 achieved their maximal effects at 1 μM (Fig. 2d). VE-822, a specific inhibitor of Ataxia Telangiectasia mutated and Rad3-related kinase (ATR), increased the efficiency by 5.9-fold compared to the control. AZD-7762, a specific inhibitor of checkpoint kinase CHEK1, also enhanced insertion efficiency by 2.7-fold. VE-822 and AZD-7762 did not significantly increase the cell proliferation rate of hPSCs, suggesting that VE-822 and AZD-7762 can increase knockin efficiency not through promoting cell proliferation (Supplementary Fig. 6a). Furthermore, hPSCs treated with VE-822 and AZD-7762 maintained the robust expression of OCT4 and NANOG (Supplementary Fig. 6b). VE-822 and AZD-7762 did not show any cytotoxicity to hPSCs (Supplementary Fig. 6c, d). Interestingly, in the context of CRISPR-Cpf1-mediated knockin in hPSCs, SCR7 did not show significant effect, but VE-822 and AZD-7762 significantly promoted CRISPR-Cpf1-mediated knockin in hPSCs by 6-fold (Fig. 2e). In the context of CRISPR-Cas9-mediated knockin, SCR7 increased the efficiency by less than 2-fold, but VE-822 and AZD-7762 worked much better and increased the efficiency by 4-fold (Supplementary Fig. 5c, d). The effects of VE-822 and AZD-7762 were replicated in multiple hESC and hiPSC lines (Fig. 2f). Knockdown of *ATR* and *CHEK1* by siRNAs could promote CRISPR-Cpf1-mediated genome editing, suggesting VE-822 and AZD-7762 work through targeting ATR and CHEK1, respectively (Fig. 2g). Sanger sequencing results demonstrated the correct targeting at the *OCT4* locus (Supplementary Fig. 5b). VE-822 and AZD-7762 could also significantly promote CRISPR-Cpf1-mediated knockin at the *ALBUMIN* locus (Supplementary Fig. 7a, b, c). Furthermore, VE-822 and AZD-7762 could significantly promote CRISPR-Cas9-mediated knockin at the *ALBUMIN* locus (Supplementary Fig. 7d, e, f). Interestingly, the effect of VE-822 and AZD-7762 was additive (Supplementary Fig. 7b, e). Using the NHEJ reporter assay^17^, we found that VE-822 and AZD-7762 did not significantly affect the NHEJ efficiency (Supplementary Fig. 8a, b, c). Taken together, we have successfully identified VE-822 and AZD-7762 that could significantly improve the CRISPR-Cpf1-mediated knockin in hPSCs.

### CRISPR-Cpf1-mediated knockin without drug selection

**Fig. 3:**
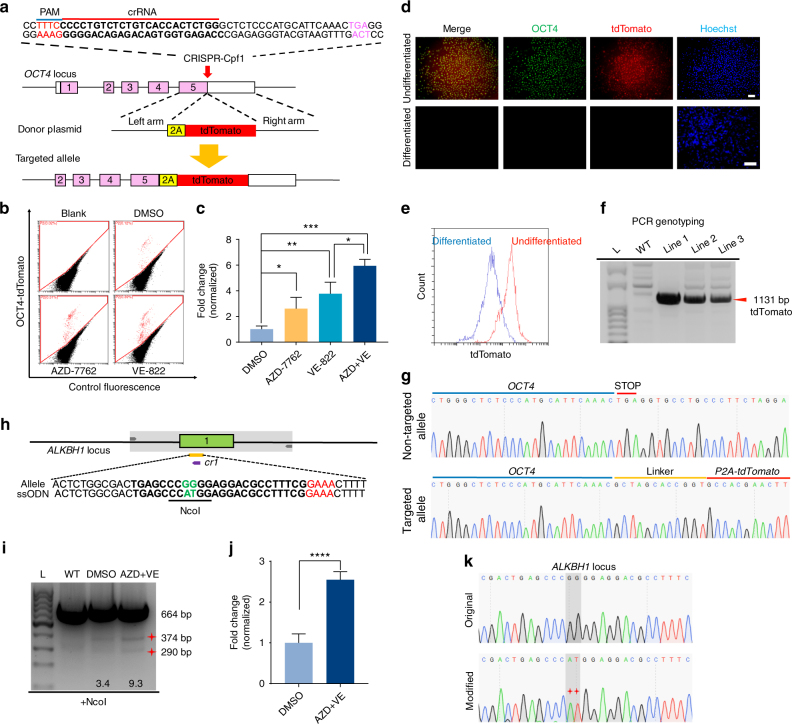
Small molecules significantly promote CRISPR-Cpf1-mediated generation of knockin hPSC lines without drug selection. **a** A scheme of the targeting strategy of OCT4-2A-tdTomato. **b** FACS analysis for OCT4-tdTomato-positive cells after transfection of the OCT4-tdTomato donor plasmid and the OCT4-targeting crRNA into hPSCs. A representative FACS result of samples treated with DMSO, VE-822, and AZD-7762 was shown. **c** Addition of VE-822 and AZD-7762 increased the percentage of OCT4-tdTomato-positive cells. *n* = 3 experiments. Statistical significance calculated using two-tailed Student’s *t*-test, compared to DMSO controls. *P < 0.05, **P < 0.01 ***P < 0.001. **d** Characterization of OCT4-tdTomato reporter lines. Scale bar, 100 μM. **e** FACS analysis of OCT4-tdTomato reporter lines. Undifferentiated line is in red, and differentiated line is in purple. **f** PCR genotyping of OCT4-tdTomato reporter lines. The size of expected bands is 1131 bp. **g** Sequencing results of the non-targeted allele and at the junction of correctly targeted allele in OCT4-tdTomato reporter lines. **h** A scheme of ssODN-mediated genome editing. We used *ALKBH1*–cr1 and a synthesized 120-nt ssODN template to introduce point mutations, which could be detected by RFLP assay. **i** The RFLP assay result. NcoI was used to evaluate the ssODN-mediated knockin efficiency. **j** VE-822 and AZD-7762 could promote ssODN-mediated genome editing by almost 3-fold. *n* = 4 experiments. Statistical significance calculated using two-tailed Student’s *t*-test, compared to DMSO controls. ****P < 0.0001. **k** Sanger sequencing confirmed the successful introduction of point mutations

Further, we examined the capacity of CRISPR-Cpf1 for the generation of knockin hPSC lines without drug selection. We co-electroporated hPSCs with three plasmids: one expressing Cpf1, one containing the specific crRNA targeting *OCT4*, and the donor plasmid OCT4-2A-tdTomato (Fig. 3a). After 5–6 days, we observed tdTomato-positive cells, and analyzed the knockin efficiency by FACS analysis. Consistently, both VE-822 and AZD-7762 could significantly improve the percentage of tdTomato-positive cells, indicating the promotion of the CRISPR-Cpf1-mediated genome editing in hPSCs (Fig. 3b, c). Interestingly, the effect of VE-822 and AZD-7762 was additive (Fig. 3c). Next, we picked several tdTomato-positive colonies for hPSC line establishment. The result of PCR analysis suggested the successful integration of tdTomato (Fig. 3f). These established hPSC lines co-expressed tdTomato with the pluripotency marker OCT4 (Fig. 3d). After directed differentiation, tdTomato expression was downregulated in the OCT4-tdTomato hPSC reporter lines with concomitant loss of endogenous OCT4 expression as determined by immunostaining and FACS analysis (Fig. 3d, e). Thus, the OCT4-tdTomato reporters faithfully reflect endogenous gene expression during the maintenance and differentiation of hPSCs. Using Sanger sequencing, we found that these OCT4-tdTomato lines correctly showed the expected sequence at the junction between the endogenous *OCT4* sequence and the inserted 2A-tdTomato sequence (Fig. 3g). In addition, we checked the effect of these small molecules on double knockin at *OCT4* and *ALBUMIN* locus. Interestingly, we found that VE-822 and AZD-7762 could significantly increase double knockin rate (Supplementary Fig. 9a, b, c), which further suggested the potential applications of these small molecules. Using genome editing tools to precisely introduce or correct point mutations has many applications. Next, we tested whether VE-822 and AZD-7762 could enhance point mutation editing using a short single-stranded oligodeoxy-nucleotide (ssODN) template. We used *ALKBH1*-cr1 and a synthesized 120-nt ssODN template to introduce point mutations, which can be detected by RFLP assay (Fig. 3h). We found that VE-822 and AZD-7762 could promote ssODN-mediated genome editing by almost 3-fold (Fig. 3i, j). Sanger sequencing confirmed the successful introduction of point mutations (Fig. 3k). These results suggested that VE-822 and AZD-7762 could also significantly promote precise genome editing using a short ssODN template. Taken together, CRISPR-Cpf1 and small molecules can be used for efficient genome editing in hPSCs.

## Discussion

It is important to develop methods for rapid, efficient, and controllable genetic manipulation of hPSCs. Here, our studies demonstrate that CRISPR-Cpf1 can be used to efficiently generate both knockout and knockin hPSC lines. CRISPR-Cpf1 not only provides an alternative method for targeted mutagenesis, but also greatly enhances the scope and precision of genome editing. Particularly, we have identified small molecules VE-822 and AZD-7762 that can significantly promote CRISPR-Cpf1-mediated hPSC genome editing by chemical screening. It is highly in demand to identify small molecules and develop chemical cocktails for precise genome editing, because multiple-site genome editing will potentially be required in many practical applications^18^. CRISPR-Cpf1 and small molecules can also be further developed and applied for in vivo genome editing and human germline genome editing^19–21^. Undoubtedly, these advances will expand the molecular toolbox of genome engineering and accelerate the development of innovative approaches for curing human diseases.

## Methods

### Plasmid construction

For pCpfcr vector, the BbsI recognized sequence and direct repeat sequence were designed on the forward primer of U6 promoter; and the U6 promoter domain was PCR amplified and then cloned into T vector by using pUCm-T Vector Cloning Kit (Sangon Biotech). For pCpfcr-crRNA vectors expressing Cpf1 target sequence, a 24-bp oligo located 3′ end of the PAM sequence was designed, annealed, and cloned into BbsI-digested pCpfcr.

For OCT4-tdTomato donor plasmid, OCT4-2A-mOrange donor plasmid (Addgene, Plasmid #66986) was PCR amplified without mOrange sequence as the backbone, and the tdTomato sequence was amplified and linked with backbone by using Gibson Assembly kit (New England Biolabs). All vectors were checked by Sanger sequencing.

### Cell culture

Human embryonic kidney 293T cells were cultured in Dulbecco’s Eagle Medium (DMEM) (Life Technologies) supplemented with 10% fetal bovine serum (Life Technologies), 100× Penicillin/Streptomycin (Life Technologies). HEK293T cells were purchased from ATCC (CRL-3216).

Human PSCs were cultured as previously reported^22^. Briefly, H1 and MEL1 hESCs and hiPSCs were maintained in hPSC medium: DMEM/F12 (Life Technologies) supplemented with 20% KnockOut Serum Replacement (Life Technologies), 1× Non-Essential Amino Acids (Life Technologies), 100× Penicillin/Streptomycin (Life Technologies), 0.055 mM 2-Mercaptoethanol (Sigma), and 10 ng mL^−1^ bFGF (Peprotech). hPSCs were dissociated by Accutase (Life Technologies) and passaged at 1:3 to 1:6 every 3–6 days. When passaging or thawing cells, 0.5 μM Thiazovivin were added into hPSC medium. H1 hESCs (NIHhESC-10-0043) were purchased from Wicell Research Institute. MEL1 hESCs were kindly provided by Drs. Ed Stanley and Andrew Elefanty. hiPSCs were produced by reprogramming human fibroblasts that were purchased from ATCC (CRL-2097).

We routinely tested the mycoplasma contamination of hPSCs.

### Electroporation

Before electroporation, all plasmids were maxiprepped using ZymoPURE Plasmid Maxiprep Kit (ZYMO RESEARCH). The electroporation solution was mixed by 81.82 μL of Solution I, 18.18 μL of Supplement I (Human Stem Cell Nucleofector^®^ Kit 1, Lonza) and plasmid mixture. For knockout experiments, plasmid mixture was prepared with 5 μg of pY016 (pcDNA3.1-hLbCpf1) (Addgene, Plasmid #69988) and 5 μg of pCpfcr-crRNA. For knockin experiments, plasmid mixture was prepared with 3 μg of pcDNA3.1-hLbCpf1, 3 μg of pCpfcr-crRNA, and 4 μg of OCT4-tdTomato donor or OCT4-2A-eGFP-PGK-Puro donor (Addgene, Plasmid #31938) or 120-nt ssODN template. For double knockin, plasmid mixture was prepared with 4 μg of pcDNA3.1-hLbCpf1, 3 μg of pCpfcr-OCT4-crRNA1, 3 μg of pCpfcr-ALBUMIN-crRNA1, 4 μg of OCT4-tdTomato donor, and 4 μg of ALBUMIN-2A-tdTomato donor. hPSCs were dissociated into single cells by using Accutase. 1 × 10^6^ cells were resuspended in electroporation solution and electroporated using Amaxa Nucleofector (Lonza) by program A-023^23^. After electroporation, cells were cultured in one well of 6-well plates for 2–3 days. Then, cells were analyzed by FACS and T7EI assays. For colony establishment, 500–2000 cells were seeded into 10-cm dishes and cultured for 4–7 days. Then, cell colonies were picked, expanded, and stocked. After 2 weeks, tdTomato-positive cell lines were analyzed by immunostaining, FACS, and Sanger sequencing.

### Transfection

70–80% confluent 293T cells in 6-well plates were transfected by using Lipofectamine^TM^ 3000 Transfection Reagent (Invitrogen) mixed with plasmid mixture. The plasmid mixture was prepared with 1 μg of pY016 (pcDNA3.1-hLbCpf1) and 1 μg of pCpfcr-crRNA. 2–3 days after transfection, cells were analyzed by T7EI assay.

### RFLP and T7EI analysis

Cells were collected 2–3 days after transfection or electroporation, and genomic DNA was extracted by using Quick-gDNA miniprep (ZYMO RESEARCH). Genomic regions containing the CRISPR target sites were PCR amplified using Taq polymerase (Vazyme).

For RFLP assay, 2 μL of PCR products were digested by restriction enzymes and analyzed by using 2% agarose gels. For point mutation, 100 μL of PCR products were cleaned and purified by using DNA Clean & Concentrator-5 (ZYMO RESEARCH) and digested by NcoI (New England Biolabs), followed by the analysis on 2% agarose gels. The knockin percentage was calculated by the formula 100 × (b + c)/(a + b + c), in which a represents the intensity of the undigested PCR product band, and b as well as c are the intensities of cleavage product bands, respectively^3^.

For T7EI assay, 10 μL of PCR products were hybridized in NEB Buffer 2 (New England Biolabs) in 16 μL total volume. The hybridization system was 95 °C for 5 min, 95–85 °C at −2 °C s^−1^, 85–25 °C at −0.1 °C s^−1^, hold at 4 °C. Next, 16 μL of products were digested by 10 U L^−1^ T7EI enzyme (New England Biolabs) in 20 μL total volume at 37 °C for 30 min, followed by the analysis on 2.5% agarose gels. The images of gels were captured by JS-2000 Gel Imager (Peiqing Science & Technology) and analyzed by ImageJ software. The indel percentage was calculated by the formula 100 × (1 – (1 – (b + c)/(a + b + c))^1/2^), in which a represents the intensity of the undigested PCR product band, and b as well as c are the intensities of cleavage product bands, respectively^3^.

### Flow cytometry

After electroporation, hPSCs transfected with Cpf1, OCT4-crRNA, and OCT4-tdTomato donor plasmids were cultured in hPSC medium for 3–4 days in one well of 6-well plates. Before FACS, undifferentiated and differentiated cells were dissociated into single cells by using Accutase for 3–5 min. Then, cells were collected and resuspended in 0.3–1 mL of 1× Dulbecco’s phosphate-buffered saline (Life Technologies). Flow cytometry data were acquired by using Beckman CytoFlex (Beckman Culture) and analyzed by CytExpert software.

### hPSC differentiation

About 200 undifferentiated OCT4-tdTomato hPSCs were seeded onto one well of 6-well plates and cultured in hPSC medium for 4–7 days. Then, hPSC medium was replaced by differentiation medium (DMEM/F12, 20% KSR, 1× NEAA, 100× Penicillin/Streptomycin (Life Technologies), 0.055 mM 2-Mercaptoethanol (Sigma), 0.1 μM LDN193189, 10 μM E616452) for 3 days. After 4–5 days, cells were analyzed by FACS and immunostaining.

### Immunostaining

Cells were fixed with 4% paraformaldehyde for 10–15 min at room temperature (RT) followed by washing with 1× PBST (1× PBS + 0.3% Triton X-100 (Vetec)) for three times with 5 min each time at RT. Then, cells were blocked in blocking buffer (1× PBST + 5% bovine serum albumin (Yeasen)) for 0.5–1 h at RT followed by the incubation with the primary antibody (anti-Nanog antibody, 1:500, ab80892, Abcam; anti-Oct4 antibody, 1:500, sc-8629, SANTA CRUZ) overnight at 4 °C. After three-time washing by PBST with 15 min each time at RT, cells were stained with the appropriated secondary antibodies for 1 h at RT. Finally, 10-min incubation with Hoechst (1:5000) was used to stain nuclei.

### Chemical screening

Kinase Inhibitor Library (MedChem Express) containing around 600 compounds was used for screening. Before electroporation, 0.2 μL of each small molecule (with a dilution of 1:1000) was added into each well of 48-well plates with 100 μL of hPSC medium. After electroporation, 1 × 10^6^ cells in 4.8 mL of hPSC medium with Thiazovivin were seeded into each 48-well plate. Cells were incubated in hPSC medium with small molecules for the first 2 days followed by hPSC medium with 1 μg mL^−1^ puromycin for 4–5 days. Then puromycin-resistant colonies were counted to quantify the effects of compounds.

### Cell apoptosis assay

Cell apoptosis was assayed by the cell apoptosis assay kit. Briefly, hPSCs were treated with DMSO, AZD-7762 (1 μM) and VE-822 (1 μM), respectively. 5 × 10^5^ cells were collected and washed twice by cold PBS. Cells were centrifuged at 300 × *g* for 5 min at 4 °C. Then, cells were resuspended in 50 μL of 1× Binding Buffer and incubated with 2.5 μL of Annexin V (AV)-FITC and 2.5 μL of PI staining Solution at RT for 10–15 min. 250 μL of 1× Binding Buffer was added into the mixture and flow cytometry was used to measure cell apoptosis.

### Off-target analysis

Potential off-target sites were found by using Cas-OFFinder^24^. The mismatches were no more than 6. The off-target sites were amplified by PCR and sequenced by Sanger Sequencing.

### NHEJ reporter assay

hPSCs were electroporated with a plasmid mixture of 4 μg of I-SceI expression vector and 4 μg of NHEJ-GFP vector^17^. 48 h after electroporation, the percentage of GFP-positive cells was determined by FACS.

### Statistics

Indicated *P*-values were obtained using a two-tailed *t*-test, and all quantitative data are shown as mean ± s.e.m. No statistical method was used to predetermine sample size. No samples were excluded. The experiments were not randomized. The investigators were not blinded to allocation during the experiments and outcome assessment.

### Data availability

Data are available from the authors upon reasonable requests.

## Electronic supplementary material

Supplementary Information(PDF 30248 kb)
