# Supplementary material for: Small molecules promote CRISPR-Cpf1-mediated genome editing in human pluripotent stem cells
Source: Nat Commun. 2018 Apr 3;9:1303. doi: 10.1038/s41467-018-03760-5 (PMC5880812; doi:10.1038/s41467-018-03760-5)
Supplement: Supplementary file 1 — Supplementary Information(PDF 30248 kb) [file 41467_2018_3760_MOESM1_ESM.pdf]

SUPPLEMENTARY INFORMATION

Small molecules promote CRISPR-Cpf1-mediated genome editing in human pluripotent stem cells

Ma et al.

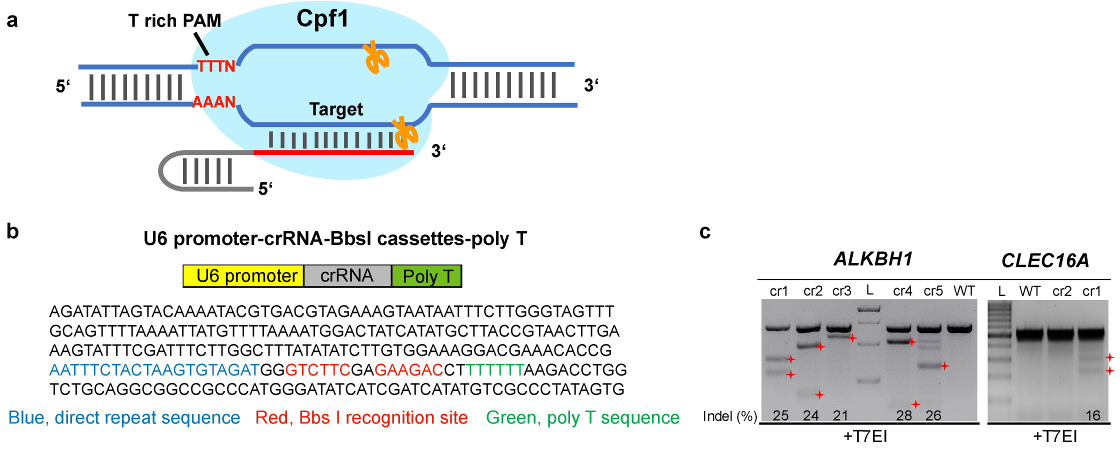

Supplementary Figure 1. (a) A brief description of the properties of CRISPR-Cpf1-based genome editing system. **(b)** The construction of pCpfcr vector. The direct repeat sequence (blue), BbsI recognized sequence (red) and poly T sequence (green) were followed after U6 promoter. **(c)** T7EI assay for crRNAs of *ALKBH1* and *CLEC16A* tested in 293T cells. The Indel frequency was calculated using the expected fragments.

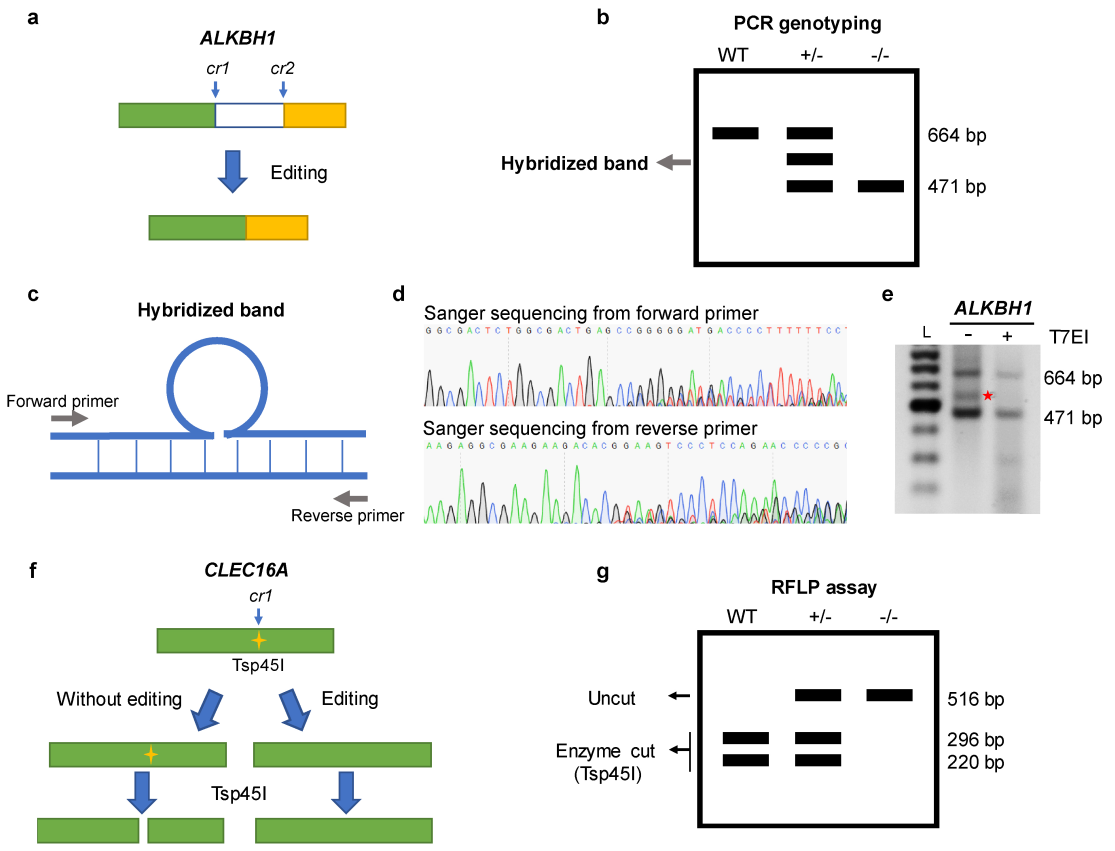

Supplementary Figure 2. (a) For knockout of *ALKBH1*, ALKBH1-crRNAs cr1 and cr2 were used. If two crRNAs worked successfully, there was about 190 bp deletion at *ALKBH1* locus. **(b)** A description of PCR genotyping result. A 471-bp band for homozygous *ALKBH1* knockout clones, and a 664-bp band for WT clones. In heterozygous *ALKBH1* knockout clones, we observed two bands around 471 bp and 664 bp, and an additional hybridized band. **(c)** A brief description of the hybridized band. **(d)** The Sanger sequencing results of the hybridized band. **(e)** A T7EI assay of the hybridized band. The hybridized band could be specifically cut by T7EI. **(f)** A brief description of RFLP assay of *CLEC16A* knockout. The CLEC16A-cr1-targeting site contains Tsp45I site. **(g)** A description of RFLP result. For WT clones, the Tsp45I restrictive enzyme site was intact, and there were two bands (220 bp and 296 bp). For homozygous *CLEC16A* knockout clones, the Tsp45I site was edited with Indels. Therefore, we could obtain a larger band with 516 bp. For heterozygous *CLEC16A* knockout clones, there were three bands (220 bp, 296 bp and 516 bp).

a

Target gene and crRNA	crRNA sequence (PAM sequence in red)	Potential off-target	Mismatch	Potential off-target sequence	Chromatin	Site
ALKBH1-cr1	TTTCCGAAAGGCGTCCTCCCCGGGCTCA	*CERCAM*	5	TTTTaGAAAGGCGTgCTggCCGGaCTCA	9	131177350
		FAM219A	5	TTTGgaAAAGGCtTCCTCCCttGGCTCA	9	34408567
		PALLD	6	TTTTacAAAtcCaTCCTCCCaGGGCTCA	4	169509348
		AHCYL2	6	TTTTCcAgAGGCtTCCaCCCaGtGCTCA	7	129035595
ALKBH1-cr2	TTTGCCCCGCGTCTTGGAGGGACTTACG	*ZDHHC3*	5	TTTCCCtCcCtTCTTGGAGGGACTTgtG	3	44968529
		DNAJB6	6	TTTTCCCCGtcTCTaGaAGGGtCTTAgG	7	157151017
		TGM3	6	TTTTCtCCttGTCTTGGgGGGACTTAaG	20	2279886
		TST	6	TTTGCCtCcCtTCTTGGAGGGAgTgAgG	22	37411362

b

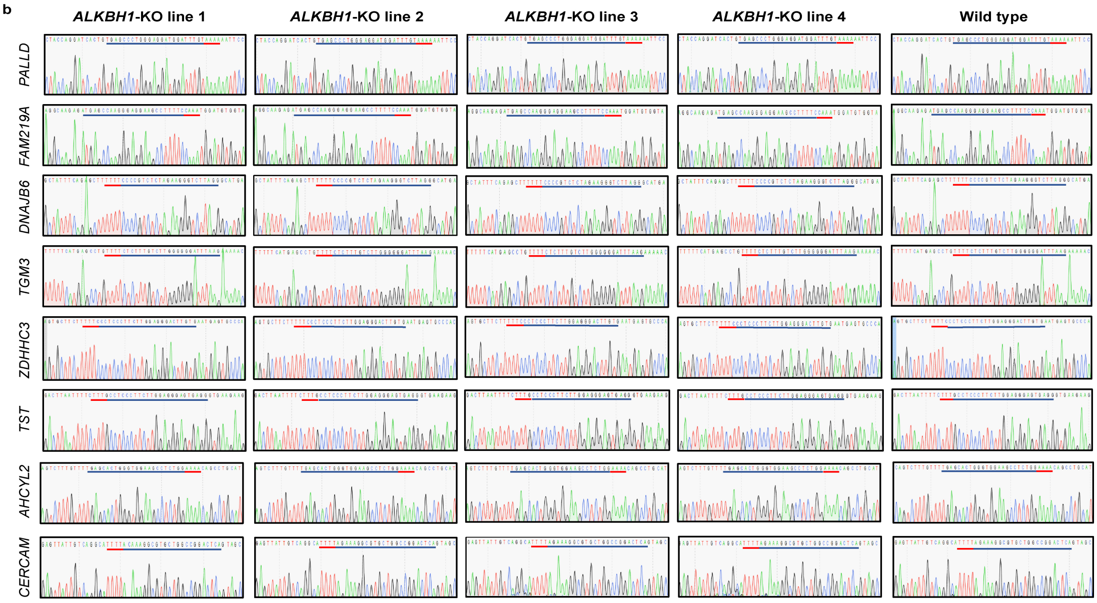

Supplementary Figure 3. (a) Detailed information of potential off-target analysis of *ALKBH1* knockout lines. **(b)** The Sanger sequencing results of potential off-target sites. Briefly, potential off-target sites were amplified by PCR and sequenced by Sanger sequencing.

a

Target gene and crRNA	crRNA sequence (PAM sequence in red)	Potential off-target	Mismatch	Potential off-target sequence	Chromatin	Site
CLEC16A-cr1	TTTGACCAAAAACACCACAGTCACAGAA	*CLVS2*	4	TTTGACCAAAAACAaaACAGTaACAaAA	6	123356268
		GABBR1	5	TTTTACCAAAAAgtgaACAGTCACAGAc	6	29553273
		THSD7B	5	TTTCAttAAAAACAtCAaAcTCACAGAA	2	137578670
		TRAPPC12	5	TTTAACCAAAAAaACaACAcaCAaAGAA	2	3429692
		JAZF1	6	TTTCcaaAAAAACAggACAGTCACAGcA	7	27948247

b

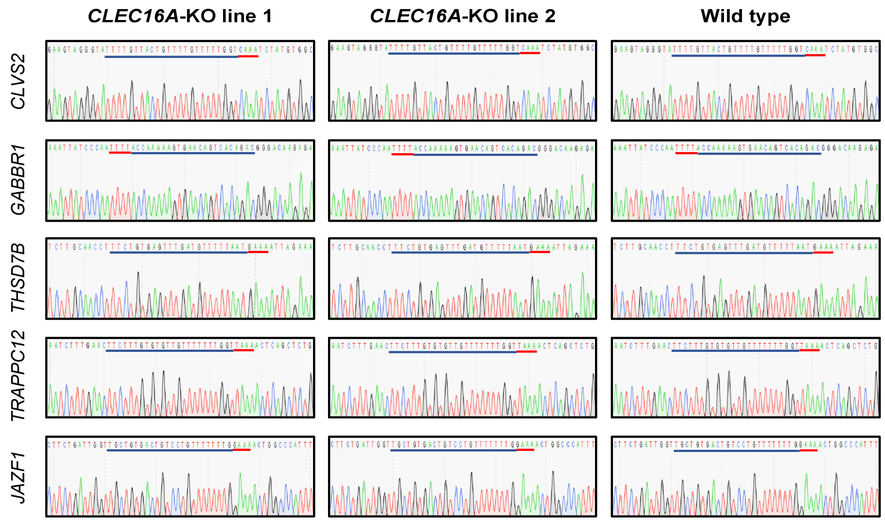

Supplementary Figure 4. (a) Detailed information of potential off-target analysis of *CLEC16A* knockout lines. **(b)** The Sanger sequencing results of potential off-target sites. Briefly, potential off-target sites were amplified by PCR and sequenced by Sanger sequencing.

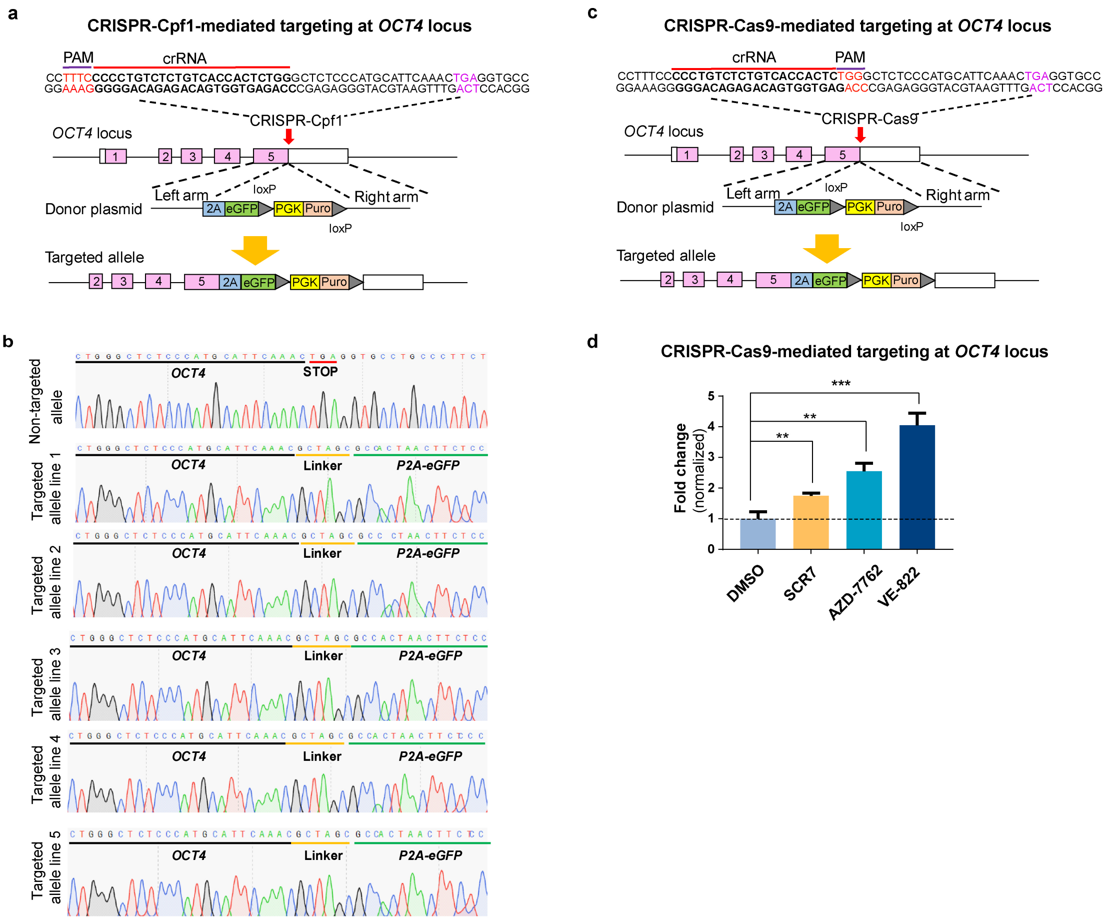

Supplementary Figure 5. (a) A scheme of the CRISPR-Cpf1-mediated targeting strategy using OCT4-2A-eGFP-PGK-Puro. OCT4 crRNA targets a sequence (indicated with a red line) downstream of the PAM sequence (in red). **(b)** Sanger sequencing results of the targeting site at the *OCT4* locus. **(c)** A scheme of CRISPR-Cas9-mediated targeting at the *OCT4* locus. **(d)** The effects of VE-822, AZD-7762 and SCR7 in context of CRISPR-Cas9-mediated knockin in hPSCs. n=3 experiments. Statistical significance calculated using two-tailed student's t-test, compared to DMSO controls. **P<0.01, ***P<0.001.

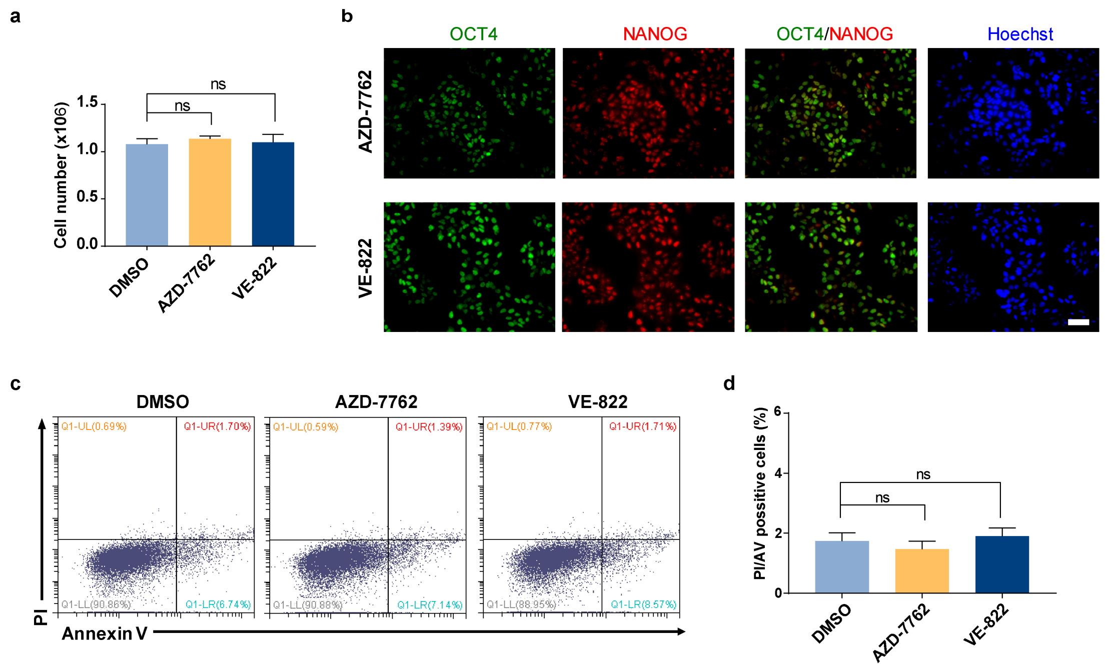

Supplementary Figure 6. (a) Cell number among hPSCs treated with DMSO, AZD-7762 and VE-822. n=3 experiments. Statistical significance calculated using two-tailed student's t-test, compared to DMSO controls. **(b)** Immunostaining of hPSCs treated with VE-822 and AZD-7762. Scale bar, 100 μm. **(c)** The FACS results of cell apoptosis. Cell apoptosis was assayed by the cell apoptosis assay kit. **(d)** The percentage of apoptosis cell population treated with AZD-7762 and VE-822. n=3 experiments. Statistical significance calculated using two-tailed student's t-test, compared to DMSO controls.

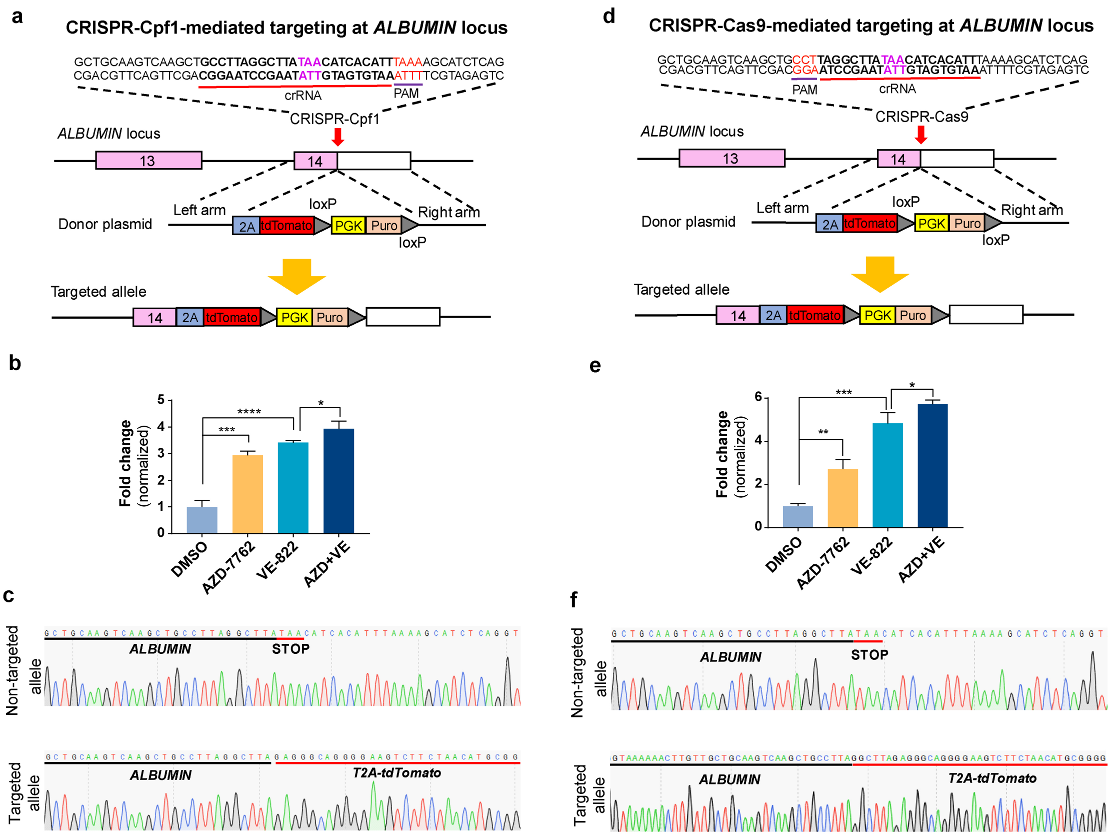

Supplementary Figure 7. (a) A scheme of CRISPR-Cpf1-mediated targeting at *ALBUMIN* locus. **(b)** The effects of VE-822 and AZD-7762 in context of CRISPR-Cpf1-mediated knockin at *ALBUMIN* locus. n=3 experiments. Statistical significance calculated using two-tailed student's t-test, compared to DMSO controls. *P<0.05, ***P<0.001, ****P<0.0001. **(c)** Sanger sequencing results of the targeting site at *ALBUMIN* locus. **(d)** A scheme of CRISPR-Cas9-mediated targeting at *ALBUMIN* locus. **(e)** The effects of VE-822 and AZD-7762 in context of CRISPR-Cas9-mediated knockin at *ALBUMIN* locus. n=3 experiments. Statistical significance calculated using two-tailed student's t-test, compared to DMSO controls. *P<0.05, **P<0.01, ***P<0.001. **(f)** Sanger sequencing results of the targeting site at *ALBUMIN* locus.

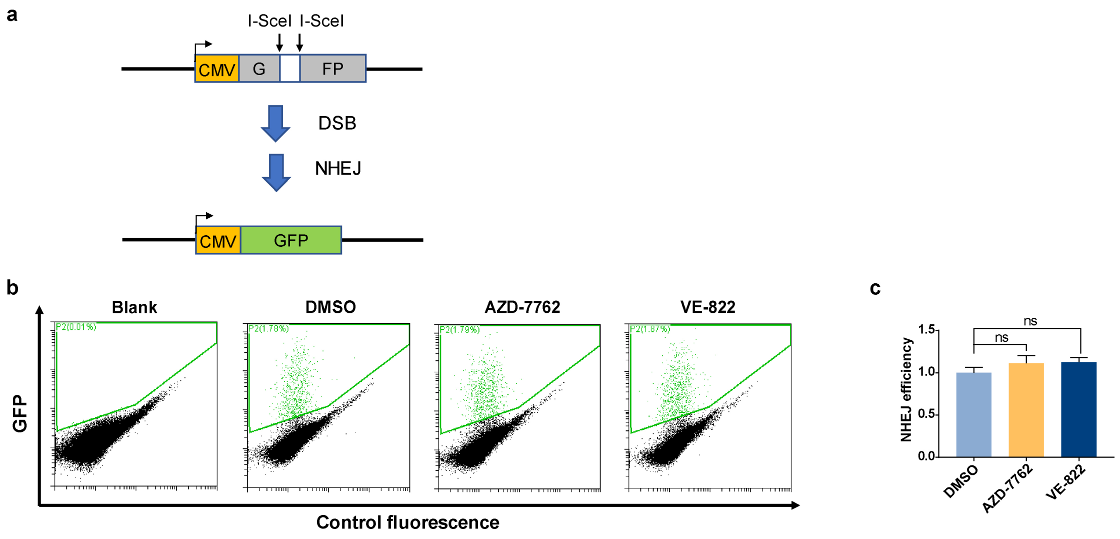

Supplementary Figure 8. (a) A scheme of the NHEJ reporter assay. **(b)** The FACS results of NHEJ reporter assay. **(c)** The NHEJ efficiency after the treatments of VE-822 and AZD-7762. n=3. Statistical significance calculated using two-tailed student's t-test, compared to DMSO controls.

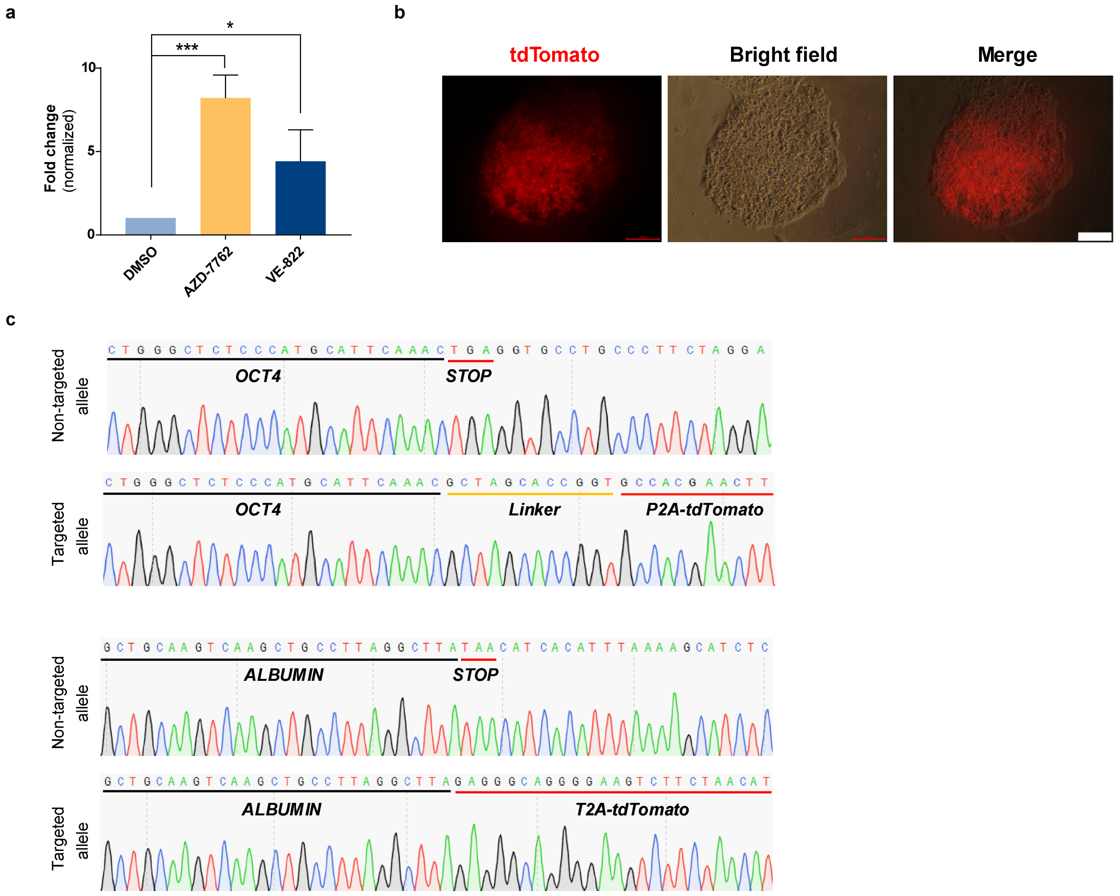

Supplementary Figure 9. (a) The effects of VE-822 and AZD-7762 in the context of CRISPR-Cpf1-mediated double knockin at *OCT4* and *ALBUMIN* locus. n=3 experiments. Statistical significance calculated using two-tailed student's t-test, compared to DMSO controls. *P<0.05, ***P<0.001. **(b)** A representative tdTomato-positive colony after double knockin. Scale bar, 100 µm. **(c)** Sanger sequencing results of the targeting sites at *OCT4* and *ALBUMIN* locus.

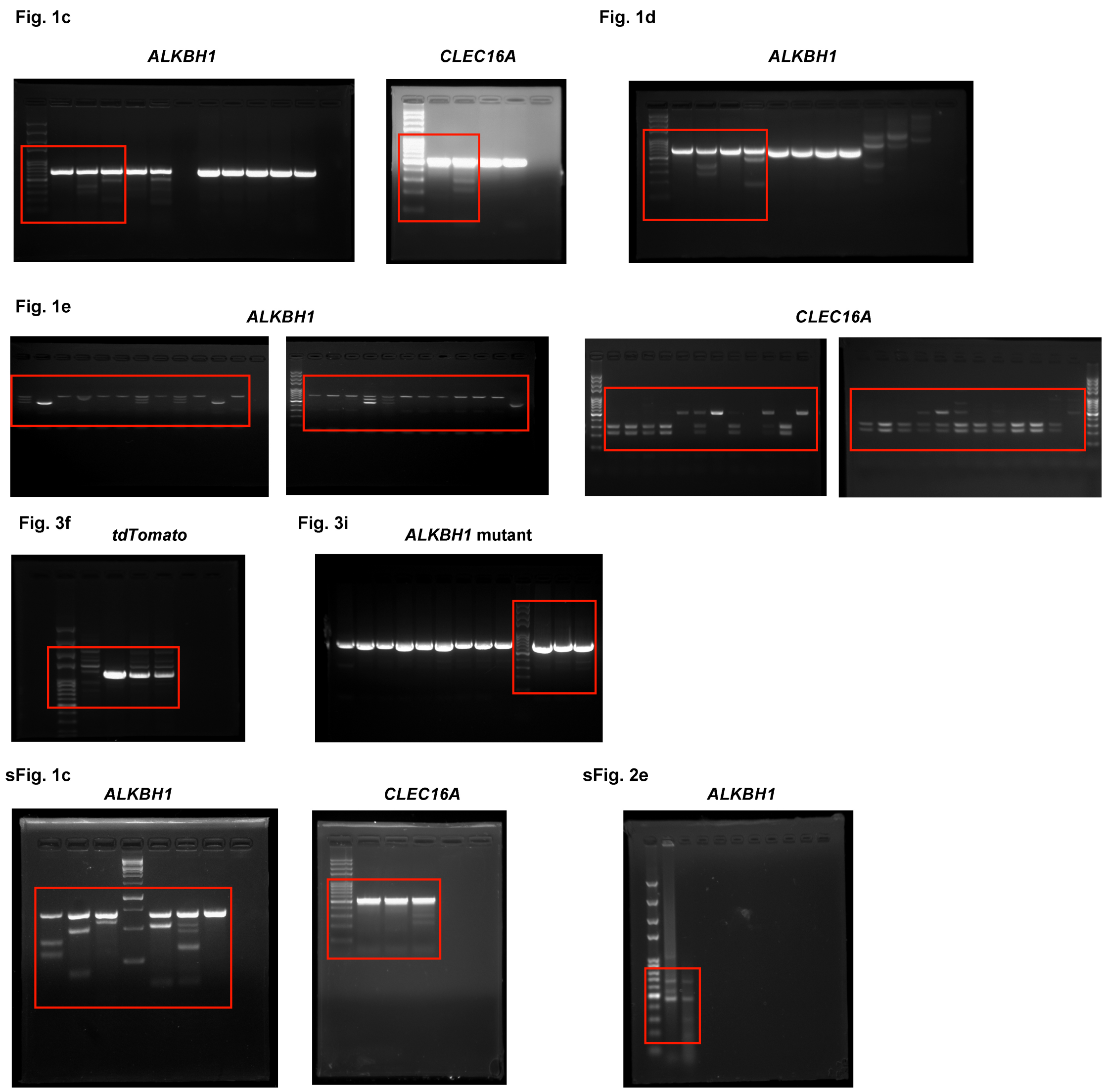

Supplementary Figure 10. Uncropped images of gels.

Table 1. Oligonucleotides used in this study

Oligonucleotides for constructing crRNA expressing plasmids

Gene		CRISPR target sequence (5' to 3', PAM in red)	Vector	Oligos used for generating the CRISPR constructs (5' to 3')
OCT4	cr1	TTTCCCCCTGTCTCCGTCACCACTCTGG	pCpfcr	F: AGATCCCCTGTCTCTGTCACCACTCTGG<br>R: AAAACCAGAGTGGTGACAGAGACAGGGG
	cr1 (for Cas9)	CCCTGTCTCCGTCACCACTCTGG	PX459	F: CACCGCCCTGTCTCCGTCACCACTC<br>R: AAACGAGTGGTGACGGAGACAGGGC
ALKBH1	cr1	TGAGCCCGGGGAGGACGCCTTTCGGAAA	pCpfcr	F: AGATCGAAAGGCGTCCTCCCCGGGCTCA<br>R: AAAATGAGCCCGGGGAGGACGCCTTTCG
	cr2	TTTGCCCCGCGTCTTGGAGGGACTTACG	pCpfcr	F: AGATCCCCGCGTCTTGGAGGGACTTACG<br>R: AAAACGTAAGTCCCTCCAAGACGCGGGG
	cr3	CCCACTAACCGTCACACTTAGCAATAAA	pCpfcr	F: AGATTTGCTAAGTGTGACGGTTAGTGGG<br>R: AAAACCCACTAACCGTCACACTTAGCAA
	cr4	TTTCACCTCCAGACCGATTGTTATTGAA	pCpfcr	F: AGATACCTCCAGACCGATTGTTATTGAA<br>R: AAAATTCAATAACAATCGGTCTGGAGGT
	cr5	TTTCGGAAACTTTTCCGCTTCTACCGTC	pCpfcr	F: AGATGGAAACTTTTCCGCTTCTACCGTC<br>R: AAAAGACGGTAGAAGCGGAAAAGTTTCC
CLEC16A	cr1	TTTGACCAAAAACACCACAGTCACAGAA	pCpfcr	F: AGATACCAAAAACACCACAGTCACAGAA<br>R: AAAATTCTGTGACTGTGGTGTTTTTGGT
ALBUMIN	cr1	GCCTTAGGCTTATAACATCACATTTAAA	pCpfcr	F: AGATAATGTGATGTTATAAGCCTAAGGC<br>R: AAAAGCCTTAGGCTTATAACATCACATT
	cr1 (for Cas9)	CCTTAGGCTTATAACATCACATT	PX459	F: CACCGAATGTGATGTTATAAGCCTA<br>R: AAACTAGGCTTATAACATCACATTC

PCR primers for plasmid construction

Primer name	Primer sequence (5' to 3')
U6-F	GAGGGCCTATTTCCCATGATTCCT
pCpfcr-R	AAAAAAAGGTCTTCTCGAAGACCCATCTACACTTAGTAGAAATTCGGTGTTTCGTCCTTTCCACAAG
mOrange-F	GGTGCCTGCCCTTCTAGGAA
mOrange-R	AGGACCGGGGTTTTCTTCCA
tdTomato-F	TGGAAGAAAACCCCGGTCCTATGGTGAGCAAGGGCGAGGA
tdTomato-R	TTCCTAGAAGGGCAGGCACCTTACTTGTACAGCTCGTCCA

PCR primers for genotyping and Sanger sequencing

Gene	Primer sequence (5' to 3')
OCT4	OCT4-tdTomato-F: ATCTTCAGGAGGTAAGGGTG<br>tdTomato-R: CGATCTCGAACTCGTGGC
	OCT4-eGFP-F: GCTTCCATCACTGGCTCGTA<br>OCT4-eGFP-R: CGACATCCCCTGCTTGTTTC
ALKBH1	F: AAATTCCACTACTCCCACATCTCC

	R: CGGATTCCCAGGCTCTTTTG
CLEC16A	F: ACCCTTCAAAGCATTGTCTGC
	R: AACTTCCCCATTTTTGGCTTG
ALBUMIN	F: ATGTCCGTGAGCTTCCGTC
	tdTomato-R: CGATCTCGAACTCGTGGC

PCR primers for T7EI assay

Gene	Primer sequence (5' to 3')
OCT4	F: GATCAAGCAGCGACTATGCA
	R: TCACTTGGGTATGAGCATTG
ALKBH1	F: AAATTCCACTACTCCCACATCTCC
	R: CGGATTCCCAGGCTCTTTTG
CLEC16A	F: ACCCTTCAAAGCATTGTCTGC
	R: AACTTCCCCATTTTTGGCTTG

PCR primers for RFLP assay

Gene	Primer sequence (5' to 3')
ALKBH1	F: AAATTCCACTACTCCCACATCTCC
	R: CGGATTCCCAGGCTCTTTTG
CLEC16A	F: ACCCTTCAAAGCATTGTCTGC
	R: AACTTCCCCATTTTTGGCTTG

PCR primers for qPCR

Gene	Primer sequence (5' to 3')
CHEK1	F: ATATGAAGCGTGCCGTAGACT
	R: TGCCTATGTCTGGCTCTATTCTG
ATR	F: GGCCAAAGGCAGTTGTATTGA
	R: GTGAGTACCCCAAAAATAGCAGG
GAPDH	F: TGCACCACCAACTGCTTAGC
	R: GGCATGGACTGTGGTCATGAG

PCR primers for off-target analysis

Gene	Primer sequence (5' to 3')
PALLD	F: GGCGTGTCACAAGAAGGG
	R: GGAGGCGGAGGATGAAGT
DNAJB6	F: TGTGGGTGACAGAATGGT
	R: AATGGGTCCTGCAAGTAA
ZDHHC3	F: CACGCCAAAGCCTTCATA
	R: GAACGGATGGGACGGTAG
AHCYL2	F: TTAGGCAGACCTCTGTGA
	R: TGGGCAACAAGAGCAAAG
FAM219A	F: ATTGAAACGCCACAGATT
	R: CTAGCCCTTAGCCTTGAC

TGM3	F: CGTATTTCAGCGTTTGTTC<br>R: GCTAAGCGAGATTTCCTAA
TST	F: CCCTAAATGGAGCGAGAA<br>R: GTAGCCTTGGGAAACAGC
CERCAM	F: CATAAGGTCGGGTCAGGG<br>R: AAGAAAGCACCAAGGCAC
CLVS2	F: ACAGAGTATCGCAGGCACAA<br>R: CCTTTCCACCCACCCTTGAT
GABBR1	F: GTGACTGTTTCCCTTTCCCTCT<br>R: TGTCATGGGGAATTGCTGGT
THSD7B	F: GAATTTAGGTGAAGGGTA<br>R: ACAAGTCGAGCTGATACA
TRAPPC12	F: GTTGTCAGGAAATGCAGAGCC<br>R: AGTCCTACGTCCGAGAGTGA
JAZF1	F: ACATATTCAACAGGTGCCAGC<br>R: AAGTCTCATGGGTTTTGGGGG

ssODN template

Gene	Sequence (5' to 3')
ALKBH1	GCGAGATGGGGAAGATGGCAGCGGCCGTGGGCTCTGTGGCGACTCTGGCGACTGAGCCCATGGAGGACGCCTTTCGGAAACTTTTCCGCTTCTACCGTCAGAGCCGGCCCGGGACCGCAG
